# Allergen specific immunotherapy enhanced defense against bacteria via TGF-β1-induced CYP27B1 in asthma

**DOI:** 10.18632/oncotarget.19826

**Published:** 2017-08-02

**Authors:** Junyi Wang, Xiaoyu Liu, Hui Wang, Yin Li, Nan lan, Xiefang Yuan, Min Wu, Zhigang Liu, Guoping Li

**Affiliations:** ^1^ Inflammation & Allergic Diseases Research Unit, Affiliated Hospital of Southwest Medical University, Luzhou 646000, China; ^2^ The State Key Laboratory of Respiratory Disease for Allergy at Shenzhen University, Shenzhen University School of Medicine, Shenzhen 518060, China; ^3^ The First Clinic College, Chongqing Medical University, Chongqing 401331, China; ^4^ Department of Biomedical Sciences, University of North Dakota, Grand Forks, North Dakota 58203, United States of America; ^5^ Department of Respiratory Disease, the Third People’s Hospital of Chengdu, Chengdu 610031, China

**Keywords:** asthma, specific immunotherapy, innate immunity, cathelicidin, TGF-β

## Abstract

Allergen specific immunotherapy (SIT) is the only specific treatment of allergic diseases at present. How SIT impacts pulmonary innate immunity against bacteria currently remains unclear. In this study, dust mite extracts (HDM)-sensitized mice were immunized with a subcutaneous injection of HDM. These mice were then challenged with an intranasal administration of HDM. After the last challenge, mice were infected with an intranasal instillation with *P. aeruginosa (P.a).* We measured the score of tissue inflammation, the expression of cathelicidin-related antimicrobial peptide (CRAMP) and 25-Hydroxyvitamin D-1Alpha-hydroxylase (CYP27B1) in lung. We analyzed the effect of TGF-β1 on CRAMP and CYP27B1 in airway cells (16HBE), and investigate the role of TGF-β1-induced CYP27B1 in defense against bacteria in16HBE cell. We found that SIT attenuates HDM-induced airway inflammation and airway responsiveness (AHR), which is involved in the increased levels of HDM-specific IgG2a, IL-10, TGF-β1, IFN-γ, CRAMP and CYP27B1. SIT ameliorates pulmonary infectious inflammation associated with an improving defense of HDM-challenged mice against *P. aeruginosa.* Meanwhile, TGF-β1 significantly increased the expression of CYP27B1 in a dose-dependent manner. TGF-β1 did not increase the levels of CRAMP in airway epithelial cells. Furthermore, 25-dihydroxyvitamin D3 (25VD_3_) is required for TGF-β1-induced CRAMP in airway epithelial cells. CRAMP was significantly increased in TGF-β1/25VD_3_-treated 16HBE cells. These findings illustrated that TGF-β1 is a major player against bacterial infections in SIT models via induction of CYP27B1 rather than CRAMP. Collectively, these findings highlight a role for SIT enhancing host defense against bacteria depending on TGF-β1-induced CYP27B1in asthma.

## INTRODUCTION

Asthma is a chronic airway inflammation disease and represents a significant health problem worldwide [1]. Airway colonization of potentially pathogenic microorganisms in asthma is associated with severe airway obstruction, neutrophilic airway inflammation, and decreased responses to current asthma therapies [2]. Bronchial asthma can weaken innate immunity of the airway, therefore increasing the risk of lung infection [3]. In turn, bacterial infection can also aggravate bronchial asthma^2^. However, immune responses to airborne pathogens, such as bacteria, may contribute to resistance to allergic responses [4]. The relationship between asthma and pathogenic infection remains unclear.

Regular administration of inhaled corticosteroids (ICS) is associated with an increased risk of oropharyngeal colonization with streptococcus pneumoniae in children with asthma [5]. Our previous studies showed that inhaled budesonide suppressed pulmonary anti-bacterial defense through down-regulation of CRAMP (also named LL-37 and CAMP in human) [6], which is a critical antimicrobial peptide (AMP) for host defense. In addition, our previous studies also showed severe asthma exacerbation with vitamin D3 (VD_3_)-deficiency showed lower forced expiratory volume in one second (FEV1) with the phenotype of neutrophilic inflammation [7]. As the active form of VD_3_, 1,25VD_3_ can bind to vitamin D receptor-specifically form vitamin D response elements (VDREs), and therefore modulates the expression of cathelicidin [8]. Recent studies have demonstrated that conversion of precursor 25VD_3_ to active 1, 25 VD_3_ via vitamin D-activating enzyme CYP27B1 can stimulate expression of antibacterial CRAMP [9, 10]. Toll-like receptor (TLR) 2/1 ligand-induced CRAMP was positively associated with percent change in 25VD_3_. Intracrine conversion of precursor 25VD_3_ to active 1,25VD_3_ can promote the expression of CRAMP [10].

House dust mites (HDM) are the most important inhalant allergens and nearly 80% of asthma patients were sensitized to their allergens [11]. SIT is the only effective treatment for changing the natural course of allergic disease [12]. The mechanism of SIT remains unclear. Previous studies found that mice immunized with chlamydial major outer membrane protein mixed with cholera toxin and CpG oligodeoxynucleotide adjuvants induced a protection against respiratory challenge with Chlamydia, which was correlated with IFN-γ production [13]. SIT contributes to immune tolerance and long-term changes in the immune system by inducing regulatory T (Treg) cells and Th1 immune response [14]. However, the effect of HDM-immunotherapy on innate immunity against lung infection in asthma remains unresolved. In the current study, we evaluated the impact of HDM-immunotherapy in defense against bacteria. We show that SIT improved the defense of HDM-challenged mice against *P. aeruginosa* depending on TGF-β1-induced CYP27B1. We further find that 25VD_3_ is required for TGF-β1-induced CRAMP.

## RESULTS

### SIT attenuates HDM-induced airway inflammation

**Figure 1:**
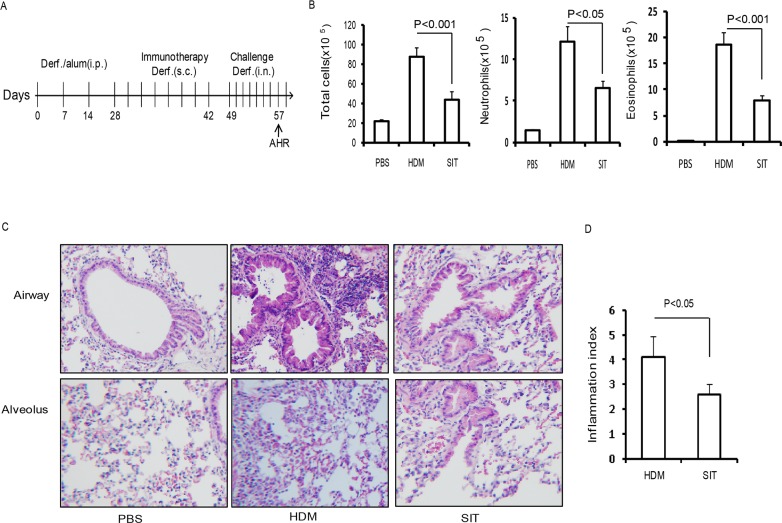
HDM-immunotherapy attenuates HDM-induced airway inflammation **(A)** HDM-specific immunotherapy protocol for HDM- inducing asthmatic mice (n = 8 mice for each group). **(B)** Total number of inflammatory cells as well as eosinophils, neutrophils and lymphocytes in the BAL of mice were determined by differential cell analysis. **(C)** Lung tissues were stained using H&E (original magnification, ×200). **(D)** The inflammatory cell infiltration index was determined in the lungs in (C). One-way ANOVA for group comparisons. Significant differences in the mean values were defined as p < 0.05. PBS mice indicated PBS control mice. SIT mice indicated HDM-immunotherapy mice. HDM mice indicated HDM-sensitized and challenged mice.

To evaluate the impact of SIT in allergen-induced airway inflammation, HDM-sensitized mice received an immunotherapy using a subcutaneous injection of HDM (Figure 1A). The mice were then challenged by intranasal HDM instillations. The total number of cells and the number of inflammatory cells including eosinophils, neutrophils and lymphocytes in the BAL fluid were decreased in the HDM-treated mice (SIT) compared to the HDM-sensitized and challenged mice (HDM) (Figure 1B). The total number of cells showed a 51.2 percent decrease, and eosinophils showed a 63.6 percent decrease in SIT mice compared to HDM mice (Figure 1B, p<0.001 and p<0.001, respectively). Inflammatory cell infiltration into airways and alveoli was decreased in SIT mice compared to HDM mice (Figure 1C). The scores of cellular infiltration into the lungs were also significantly decreased in SIT mice compared to HDM mice (Figure 1D, P<0.05). These data showed that SIT decreased HDM-induced airway inflammation.

### SIT attenuates AHR associated with increased HDM-specific IgG2a

**Figure 2:**
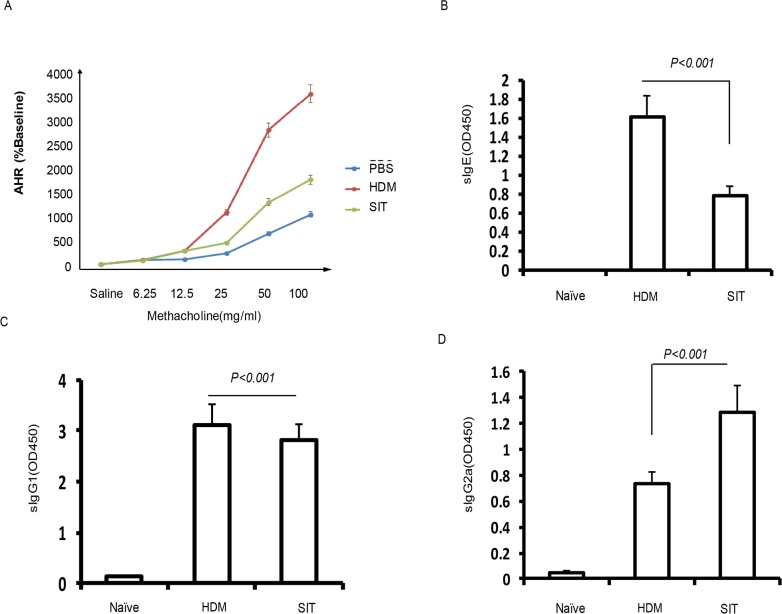
HDM-immunotherapy attenuates AHR and increased HDM-specific IgG2a **(A)** AHR was determined by measuring the enhanced pause (Penh) (n=8 each group). Serum levels of allergen-specific **(B)** IgE, **(C)** IgG1 and **(D)** IgG2a were measured by ELISA as optical density (OD). Data were shown as means±SDEV (n=8). One-way ANOVA for group comparisons. Significant differences in the mean values were defined as p < 0.05.

Previous studies have shown that AHR were strongly reduced upon ovalbumin (OVA)- immunotherapy [15]. To further investigate effect of HDM-immunotherapy on lung function in HDM mice, we measured airway responsiveness to methacholine challenges by using Buxco whole-body plethysmography system. HDM mice challenged with methacholine demonstrated methacholine dose-dependent increases in Penh levels, which were significantly reduced in SIT mice compared to HDM mice (Figure 2A, p<0.001). Having confirmed that induced IgG4 seems to suppress IgE levels, which was associated with treatment efficacy in allergic patients receiving subcutaneous immunotherapy [16]. SIT selectively removed long-lived IgE antibodies on mast cells [17]. To assess the effect of SIT in levels of antibodies to HDM, HDM-specific antibodies were measured. The HDM-specific IgE and IgG1 levels showed 51.3% and 12.5% decrease in SIT mice compared to HDM mice, respectively (Figure 2B, p<0.001; Figure 2C, p<0.001). By contrast, the HDM-specific IgG2a level showed a 1.86-fold increase in SIT mice compared to HDM mice (Figure 2D, p<0.001). These findings indicated that AHR was strongly reduced upon SIT, which was associated with increased HDM-specific IgG2a, but decreased HDM-specific IgE and IgG1.

### SIT increases the levels of IL-10, TGF-β1, and IFN-γ

**Figure 3:**
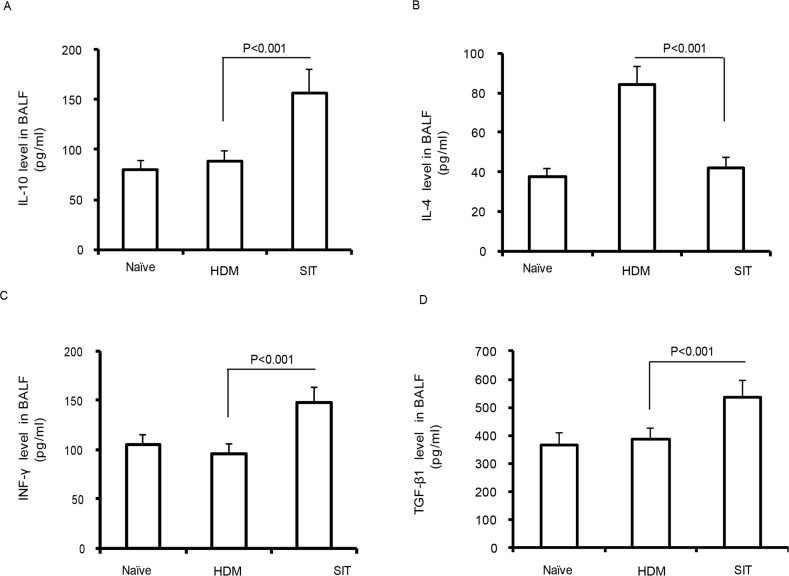
HDM-immunotherapy increases the levels of IL-10, TGF-β1, and IFN-γ Standard ELISA was performed to determine the levels of IL-10 **(A)**, IL-4 **(B)**, IFN-γ **(C)**, and TGF-β1 **(D)** in BAL fluid. Data were shown as means± SDEV (n=8). One-way ANOVA for group comparisons. Significant differences in the mean values were defined as p < 0.05.

Previous studies showed that immunotherapy ameliorated airway inflammation via IL-10 in a chronic asthma model [18]. IL-10 and TGF-β1 produced by functional Treg cells are now well established during immunotherapy [19]. We observed a robust decrease of IL-4 in SIT mice compared to the HDM mice (Figure 3A, p<0.001). By contrast, the levels of IL-10, IFN-γ and TGF-β1 showed 1.48-fold, 1.57-fold and 1.41-fold increase in SIT mice compared to the HDM mice, respectively (Figure 3B, p<0.001; Figure 3C, p<0.001; Figure 3D, p<0.001). These findings suggest that SIT increased the levels of IL-10, TGF-β1, and IFN-γ, while decreased IL-4 in HDM-induced airway inflammation.

### SIT increases the expression of CRAMP and CYP27B1 in lung

**Figure 4:**
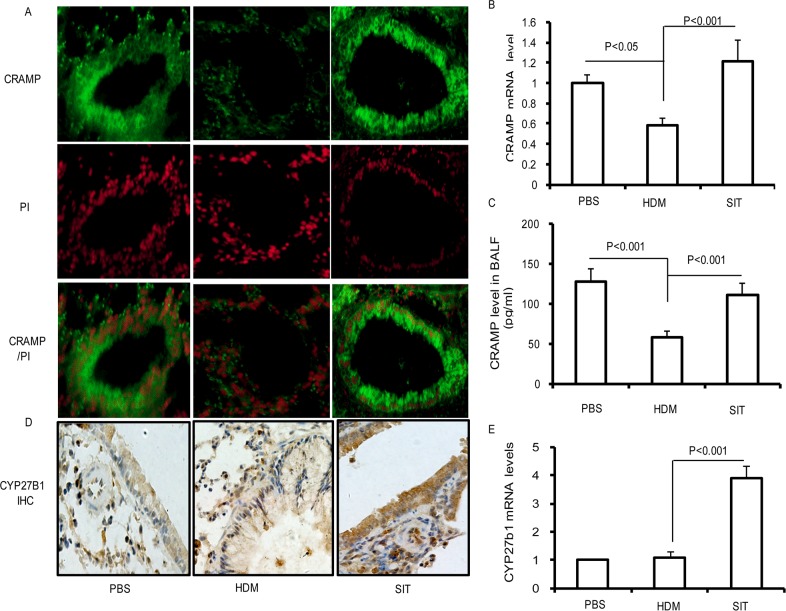
HDM-immunotherapy increases the expression of CRAMP and CYP27B1 in lung **(A)** Frozen lung sections were stained with polyclonal antibodies against CRAMP followed by incubation with FITC-conjugated secondary antibody (green) or PI (red). Sections were observed using confocal microscopy (original magnification×400). Results are representative of three experiments. **(B)** The mRNA expression of CRAMP was determined in the lungs using Real-time-PCR. **(C)** Standard ELISA was performed to determine the levels of CRAMP levels in the BAL fluid. **(D)** Lung sections were stained with polyclonal antibodies against CYP27B1. Sections were observed using microscopy (original magnification×200). **(E)** The mRNA expression of CYP27B1 was determined in the lungs using Real-time-PCR. Data were shown as means±SDEV (n=8). One-way ANOVA for group comparisons. Significant differences in the mean values were defined as p < 0.05.

Allergic individuals are more susceptible to respiratory tract infections due to an impaired antimicrobial defense, and SIT upregulates the levels of human β-defensins in patients with seasonal allergic rhinitis [16, 17]. To further investigate whether SIT improved cathelicidin (CRAMP) in HDM mice, we measured CRAMP in lungs and BALF. As seen in Figure 4A, PBS control mice showed a predominant expression of CRAMP in airway epithelium, which were significantly reduced in HDM mice. By contrast, SIT with HDM resulted in an increased expression of CRAMP in airway epithelium. To examine the transcript levels of CRAMP genes, real-time-PCR was utilized to detect the mRNA levels of CRAMP in lung tissue. The CRAMP transcription level showed a 42% decrease in HDM mice compared to PBS control mice (Figure 4B, p<0.05). As compared with HDM-exposed mice, the CRAMP transcript level showed a 2.18-fold increase in SIT mice (Figure 4B, p<0.001). In addition, we found that CRAMP protein production in BALF showed a 57% decrease in HDM mice as compared with PBS control mice (Figure 4C, p<0.001). Meanwhile, the CRAMP production in BAL fluid showed a 2.16-fold increase in SIT mice compared to HDM mice (Figure 4C, p<0.001). We further analyzed the expression of CYP27B1 in the lung with immunohistochemistry and real-time PCR. As seen in Figure 4D, the expression of CYP27B1 protein in lungs was low in PBS mice and mice exposed to HDM. However, SIT predominantly increased the expression of CYP27B1 protein in lungs. We also evaluated CYP27B1 mRNA levels in whole lungs. In PBS control mice and HDM-exposed mice, CYP27B1 mRNA levels were low. CYP27B1 mRNA levels showed a 3.8-fold increase in SIT mice compared to HDM mice (Figure 4E, p<0.001). Taken together, these findings suggested that SIT increased CRAMP gene transcript and protein production associated with the upregulation of CYP27B1 expression in mice.

### SIT ameliorates pulmonary infection inflammation

**Figure 5:**
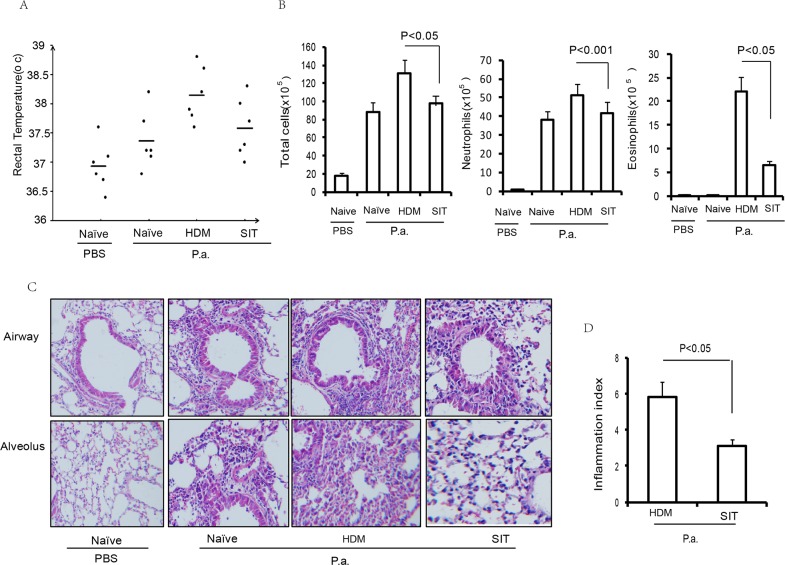
HDM-immunotherapy ameliorates pulmonary infection inflammation **(A)** Rectal temperature was recorded as part of routine monitoring for inflammation responsiveness to *P. aeruginosa* infection. Data were shown as means±SDEV (n=6). **(B)** The total number of inflammatory cells as well as eosinophils, neutrophils and lymphocytes in the BAL of mice were determined by differential cell analysis. **(C)** The lung tissues were stained using H&E (original magnification, ×200). **(D)** The inflammatory cell infiltration index was determined in the lungs in (C). One-way ANOVA for group comparisons. Significant differences in the mean values were defined as p < 0.05.

Previous studies have implicated that allergic airway inflammation suppresses the innate antimicrobial host defense [3]. However, whether SIT regulates innate antimicrobial host defense remains unclear. To investigate whether SIT improved host defense against bacteria, we evaluated inflammation responsiveness to *P. aeruginosa* infection in HDM-exposed mice. 24 hour after the last HDM challenge, temperature data were collected as part of routine monitoring for inflammation responsiveness to *P. aeruginosa* infection. All mice showed a marked increase in temperature at 24 hours after *P. aeruginosa* administration. HDM-exposed mice showed a significant increase in temperature of 1.9°C after *P. aeruginosa* administration compared to PBS control mice. SIT mice showed a significant drop in temperature of 0.7°C compared to HDM mice (Figure 5A). Meanwhile, the total number of cells and neutrophils in the BALF predominantly decreased in SIT mice compared to the HDM mice after *P. aeruginosa* administration (Figure 5B). In addition, inflammatory cell infiltration into the airways and alveoli was determined by H&E staining (Figure 5C). All mice showed a marked cellular infiltration into the lungs at 24 hours after *P. aeruginosa* administration compared to PBS control mice. The scores of inflammatory cell infiltration into the lungs showed a 51.7 percent decrease in SIT mice compared to the HDM mice after *P. aeruginosa* administration (Figure 5D, P<0.05). These findings indicate that SIT ameliorated pulmonary infection inflammatory response and inflammatory cell infiltration during HDM mice exposure to *P. aeruginosa*.

### SIT enhances defense against bacteria

**Figure 6:**
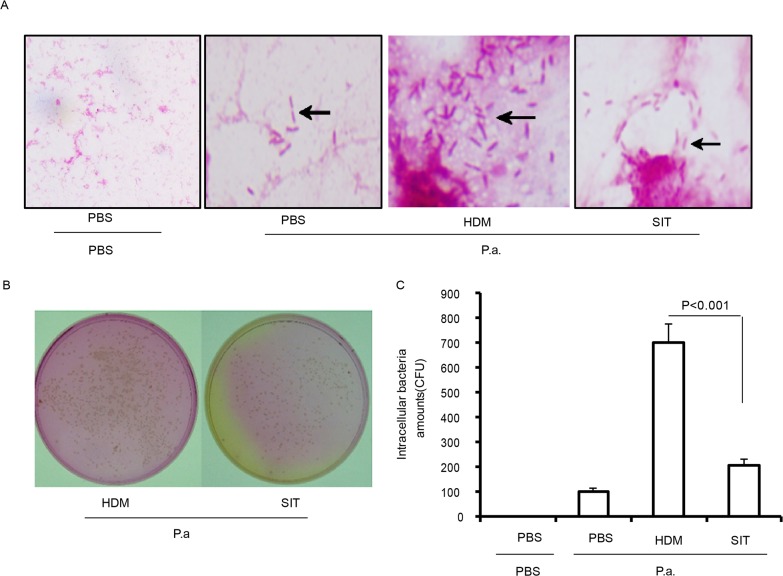
HDM-immunotherapy enhances defense against bacteria **(A)** BAL fluid were stained using a Gram’s Method. The slides were evaluated for bacteria infiltration under a light microscope (original magnification, ×200). **(B)** Represents the bacterial colonies on agar plates. **(C)** Bacterial colonies were counted in lung homogenate suspension. Data were shown as means± SDEV (n=6). One-way ANOVA for group comparisons. Significant differences in the mean values were defined as p < 0.05.

To determine whether HDM-immunotherapy can increase defense against bacteria, bacterial levels in BALF and lungs were determined at 24 h after mice exposure to *P. aeruginosa*. HDM-exposed mice showed lots of gram negative bacteria in BALF compared to PBS mice at 24 hours after *P. aeruginosa* administration. However, the number of gram negative bacteria showed a marked drop in BALF from SIT mice compared to HDM mice at 24 hours after *P. aeruginosa* administration (Figure 6A). The lung tissue of HDM mice showed a significantly increase in the number of CFU compared to PBS mice at 24 hours after *P. aeruginosa* administration (Figure 6B). The number of CFU showed a 69.4 percent decrease in lung from SIT mice compared to HDM mice at 24 hours after *P. aeruginosa* administration (Figure 6C, p<0.001). These data confirmed that SIT enhanced defense against bacteria during HDM- challenged mice exposure to *P. aeruginosa*.

### TGF-β1 increases CRAMP depending CYP27B1 in 16HBE cells

**Figure 7:**
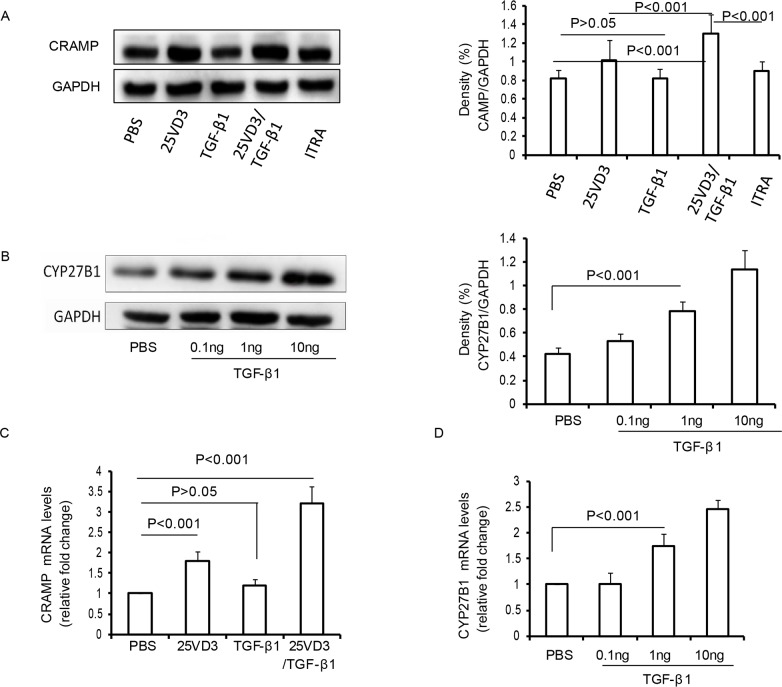
TGF-β1 increases CRAMP depending CYP27B1 in 16HBE cells **(A)** Western blot analysis of CRAMP with 16HBE cells exposed to TGF-β1, 25VD_3_, TGF-β1/25VD_3_ and ITRA/TGF-β1/25VD_3._ Relative density (change) of CRAMP in cells. β-actin was used as loading control. **(B)** Western blot analysis of CYP27B1 in 16HBE cells exposed to TGF-β1. Relative density (change) of CRAMP in cells. β-actin was used as loading control. The mRNA expression of CRAMP **(C)** and CYP27B1 **(D)** was determined in cells using Real-time-PCR. Results are representative of three experiments and data are shown as means± SDEV. One-way ANOVA for group comparisons. Significant differences in the mean values were defined as p < 0.05.

Based on our present findings that SIT with HDM associated with an increase of CRAMP and CYP27B1 in mice exposed to HDM, we analyzed the effect of TGF-β1 on CRAMP and CYP27B1 in 16HBE cell lines. We observed no change in the levels of CRAMP in TGF-β1-treated 16HBE cells compared to PBS-treated 16HBE cells (Figure 7A, p>0.05). 25VD_3_ increased the expression of CRAMP compared to PBS-treated 16HBE cells (Figure 7A, p<0.001). However, TGF-β1/25VD_3_ significantly increased the expression of CRAMP compared to 25VD_3_-treated 16HBE cells (Figure 7A, p<0.001). The chemical inhibitor of 1α-hydroxylase, ITRA, inhibited the expression of CRAMP compared to TGF-β1/25VD_3_-treated 16HBE cells (Figure 7A, p<0.001). Previous studies showed that CYP27B1 converts 25-hydroxyvitamin D_3_ into 1alpha, 25-dihydroxyvitamin D_3_ [20]. To investigate the effect of TGF-β1 on CYP27B1 in airway cells, 16HBE cells were stimulated with 0.1ng, 1ng and 10ng TGF-β1, respectively. TGF-β1 significantly increased the expression of CYP27B1 in a dose-dependent manner (Figure 7B).

To examine the transcript levels of CRAMP genes, real time-PCR was utilized to detect the mRNA levels of CRAMP. 25VD_3_ increased the transcript levels of CRAMP compared to PBS-treated 16HBE cells (Figure 7C, p<0.001), but the transcript levels for CRAMP in TGF-β1-treated 16HBE cells were unaltered, compared with PBS-treated 16HBE cells (Figure 7C, p>0.05). It was interesting that the CRAMP transcript was significantly increased in TGF-β1/25VD_3_-treated 16HBE cells compared to 25VD_3_-treated 16HBE cells (Figure 7C, p<0.001). The transcript levels of CYP27B1 was also an increased in a dose-dependent manner (Figure 7D). Taken together, these findings demonstrated that TGF-β1 did not directly increase CRAMP expression, and CYP27B1 play a key role in TGF-β1-induced CRAMP expression *in vitro*.

### TGF-β1 increases host defense against bacteria by CYP27B1-dependent activation

**Figure 8:**
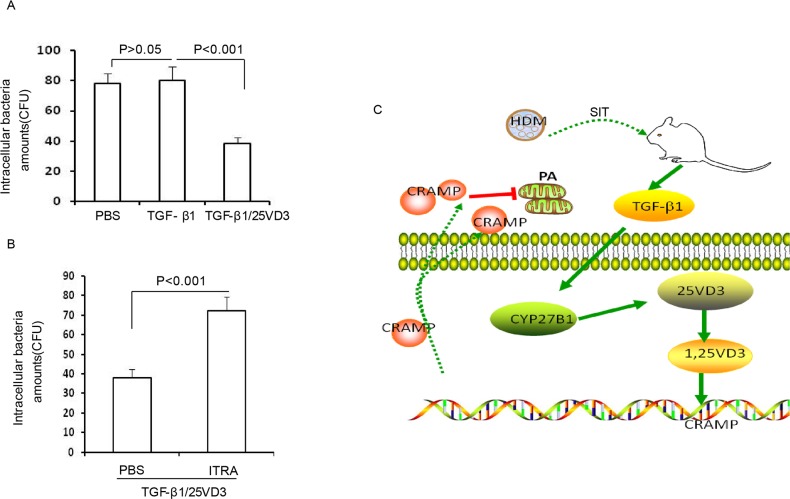
TGF-β1 increases defense against bacteria by CYP27B1-dependent activation **(A)** 16HBE cells were pretreated with TGF-β1 and TGF-β1/25VD_3_. **(B)** 16HBE cells were pretreated with ITRA. The cells infected with *P. aeruginosa* cells were lysed and plated to confirm the presence of intracellular bacteria. Results are representative of three experiments and data are shown as means±SDEV. One-way ANOVA for group comparisons. Significant differences in the mean values were defined as p < 0.05. **(C)** Schematic representation of TGF-β1 induced CYP27B1 role in HDM immunotherapy increasing defense against *P. aeruginosa* during allergic airway inflammation.

Our previous studies have confirmed that blocking CRAMP significantly increase bacterial CFUs [6]. To further investigate the effect of TGF-β1 induced CYP27B1 in defense against bacteria in airway cells, 16HBE cells were pretreated with 25VD_3_, TGF-β1, TGF-β_1_/25 VD3 followed by *P. aeruginosa* infection. The chemical inhibitor of ITRA was also used to explore the role of CYP27B1 in TGF-β1- treated 16HBE cells. We observed no change in the levels of CFUs in TGF-β1-treated 16HBE cells compared to PBS-treated 16HBE cells (Figure 8A, p>0.05). Meantime, CFUs were significantly decreased in TGF-β1/25VD_3_-treated 16HBE cells compared to 25VD_3_-treated 16HBE cells (Figure 7A, p<0.001). ITRA significantly increased the level of CFUs compared to TGF-β1/25VD_3_-treated 16HBE cells (Figure 7B, p<0.001). These data indicated that TGF-β1 increases defense against bacteria by CYP27B1-dependent activation. Figure 7F illustrates a model in which the role of HDM immunotherapy enhances host defense against bacteria during allergic airway inflammation.

## DISCUSSION

Th2 cell-mediated disorders were supposed to be the most important pathological phenomenon of allergic asthma, but it cannot fully explain the mechanism of allergic asthma. Allergen-specific immunotherapy remains as the only curative treatment. Some evidences have showed that SIT with HDM not only regulates Th2 immune response, but also modulates Treg cells [21]. The suppressor cytokines IL-10 and TGF-β produced by functional Treg cells played a key role in the generation of immune tolerance to allergens [19]. Treg cells secrete TGF-β1, which plays an important role in inhibiting allergic inflammation [22]. In our current research, an asthma mouse model was employed to detect the role of HDM-SIT. AHR of HDM-exposed mice significantly increased. The number of inflammatory cells in the BALF showed a significant increase, accompanied with more severe peribronchial and perivascular infiltration, higher IL-4 and IgE levels. HDM-SIT mice exhibited decreased lung inflammation index and airway responsiveness. The phenotype was related to the changes of IL-4, IFN-γ and IgE. Especially, Treg-cytokines IL-10 and TGF-β1 were upregulated significantly. Our experiments further confirmed that HDM-specific immunotherapy ameliorated AHR, lung inflammation and Th1/Th2 immune balance in asthma mice model, and modulated the function of Treg cells.

With low host defense against bacteria, allergic asthma patients may have increased risk of lung infection. Antibacterial peptides play an important role in innate immunity against bacteria [23]. In humans, the major classes of host defense peptides include defensins and cathelicidin [24]. CRAMP with broad spectrum microbicidal activities was expressed in airway epithelial cells, alveolar macrophages and bronchial glands, play important roles in the lung innate immune response [25]. Lung AMPs are major sentinels of innate immunity by preventing microbial colonization and infection. The bactericidal activity of AMPs against bacteria is compromised in patients with asthma [26]. Bacterial colonization was associated with initiating events of early asthma in young children with severe recurrent wheeze. Neonates colonized in the hypopharyngeal region with bacterial, are at increased risk for recurrent wheeze and asthma early in life [27]. In our present studies, the expression of CRAMP protein and mRNA in lungs was significantly reduced in HDM-exposed mice. It was further confirmed that CRAMP with asthma was inhibited. Furthermore, our studies provide the first evidence that HDM-specific immunotherapy increased the expression of CRAMP during HDM-induced allergic airway inflammation mice.

Our findings showed that HDM-specific immunotherapy increased the level of IL-10, TGF-β1, and IFN-γ, but was associated with low IL-4 in HDM-induced airway inflammation mice. It has been shown that Th2-cytokines IL-4 and IL-13 reduced antibacterial peptides against bacteria [2, 28]. IL-10 repressed the expression of anti-microbial peptide in atopic dermatitis [29]. Previous studies have also confirmed that IL-33 enhanced antimicrobial defense against skin bacterial infection via promoting antimicrobial capacity of dermal macrophages [30]. This cytokines, such as IL-17 and IL-22, induced antimicrobial proteins and neutrophil chemoattractants [31]. However, some studies showed that Type II cytokines such as IL-4 impair host defense against an intracellular fungal pathogen by amplifying macrophage generation of IL-33. IFN-γ treatment of human macrophages did not demonstrate antimicrobial activity against intracellular M. tuberculosis. Vitamin D is required for IFN-γ–mediated antimicrobial activity of human macrophages [32]. In our present study, we first demonstrated that HDM-specific immunotherapy ameliorated pulmonary infection inflammatory response and inflammatory cells infiltration after HDM-challenged mice exposure to *P. aeruginosa*. HDM-immunotherapy mice significantly reduced lung inflammation, bacterial load and rectal temperature. Therefore, SIT with HDM can indeed enhance host defense to resist respiratory tract infection (Figure 5).

Vitamin D3 has been identified to be an inducing factor of cathelicidin [8, 33, 34]. It has been confirmed that the promoter region of cathelicidin has a vitamin D response element (VDRE) sequence. Lower expression of the VDR or vitamin D3 deficiency impaired immune response [35, 36]. CYP27B1 hydroxylase enzyme is responsible for the conversion of the inactive vitamin D3 (25VD_3_) preform to the active form (1,25VD_3_) [37]. Our data showed that the expression of CYP27B1 protein in lungs was inhibited in HDM-induced airway inflammation mice. HDM-specific immunotherapy predominantly increased the expression of CYP27B1 protein in lungs (Figure 4).

HDM-specific immunotherapy predominantly increased the level of TGF-β1 in HDM-induced airway inflammation (Figure 3). However, no previous study had linked TGF-β1 to host defense against bacteria via alpha-hydroxylase, but available databases led us to consider this possibility [32, 38]. We evaluated the relevance of TGF-β1 and defense against bacteria *in vitro*. Our findings demonstrated that TGF-β1 did not directly increase CRAMP expression. Meanwhile, TGF-β1 can induce CYP27B1 in a dose dependent manner. CYP27B1 blocker itraconazole suppressed TGF-β-induced cathelicidin. Furthermore, we next investigated the effect of TGF-β1 induced CYP27B1 in defense against bacteria in airway cells. Our studies exhibited that TGF-β1/25VD_3_ significantly decreased the levels of CFUs in 16HBE exposes to *P. aeruginosa.* ITRA significantly increased the level of CFUs compared to TGF-β1/25VD_3_-treated 16HBE cells. We report for the first time that allergen-specific immunotherapy enhances defense against bacteria via TGF-β1 induced CYP27B1 during allergic airway inflammation.

In conclusion, we have found that HDM-immunotherapy decreased HDM-induce airway inflammation and increased the expression of CRAMP and CYP27B1 expression in mice. We further showed that HDM-immunotherapy enhanced defense against bacteria during HDM-challenged mice exposure to *P. Aeruginosa*. We have identified a critical role of CYP27B1 in TGF-β1-induced CRAMP expression in 16HBE cells. This study indicates that HDM-immunotherapy increased defense against *P. aeruginosa,* leading to TGF-β1-induced CRAMP expression via CYP27B1.

## MATERIALS AND METHODS

### Reagents

The CRAMP antibody(IF) (ab180760) was purchased from Abcam (Cambridge, MA, USA). The CYP27b1 antibody (sc-67261) was purchased from Santa Cruz Biotechnology (Dallas, Texas, USA). The CRAMP antibody(WB) (PAB13021) was purchased from Abnova (Taipei, Taiwan). The GAPDH antibody (Cat. No.#2118) was purchased from Cell Signaling (Danvers, MA, USA). The ELISA kits of IL-4, IL-10, IFN-gamma, and TGF-β1 were purchased from eBioscience (San Diego, CA, USA), and the ELISA kit of CRAMP was purchased from CUSABIO (Wuhan, China). The recombinant human cytokine proteins (TGF-β1, IFN-γ, and IL-10) were purchased from PeproTech (Rocky Hill, NJ, USA). 25 VD3, 1,25 VD3, and polymyxin B were purchased from Sigma-Aldrich(St. Louis, MO, USA). Itraconazole was purchase from Janssen (Beerse, Belgium). HDM extracts (*Der f* allergen) were provided by State Key Laboratory of Respiratory Disease for Allergy at Shenzhen University (Shenzhen, China)

### Immunotherapy protocol

Female BALB/c mice (4 to 6 weeks) were purchased from Guangdong Experimental Animal Center. They were maintained under specific pathogen–free conditions in the Animal Experimental Center of Shenzhen University (Shenzhen, China). All animal experiments were performed in accordance with the guidelines of the Animal Experiments Center of Shenzhen University. All experimental protocols were approved by and performed in accordance with the Medical Ethics Committee in Medical Center of Shenzhen University and the National Institute of Health guidelines on the care and use of animals. BALB/c mice were sensitized with intraperitoneal injection 100 μg of house dust mites extracts (HDM) and 2 mg of aluminum hydroxide on days 0, 7 and 14. For SIT treatment mice, HDM-treated mice were immunized with subcutaneous injection 100μg of HDM once every other day for 8 times on day 28. Meanwhile, HDM-exposed and phosphate buffered saline (PBS) control mice were immunized with subcutaneous injection of PBS. The mice were challenged with an intranasal instillation of 50 μg of HDM in 50 μl of PBS 7 times/week. Control mice were given PBS alone.

### Airway responsiveness

AHR was measured with Buxco whole-body plethysmography (WBP) system (Buxco Research Company, United States) in response to inhaled methacholine (acetyl-β-methyl-choline chloride, Sigma, The Netherlands). After the last challenge, mice were monitored for about 10 minutes in the chamber until their breathing went stable. After 5 minutes baseline was recorded, the responses were assessed for 5 minutes after inhaling different concentration of atomized methacholine solutions (0, 6.25, 12.5, 25, 50, and 100 mg/ml) respectively. About 5 minute intervals were given between tests to allow the respiratory intensity to return to the baseline. AHR was expressed as enhanced pause (Penh) as described in detail previously [39].

### Bacteria preparation

The *Pseudomonas aeruginosa* strain 103 (*P.a*.) was provided by the clinical laboratory of the first affiliate hospital of Shenzhen University. Bacteria were cultured for about 16 hours in LB medium at 37°C with shaking in 150rpm, and incubated the bacteria for about 1 hour until the mid-log phase in 10 ml fresh LB broth. Optical density (OD) was measured at 600 nm. The bacteria were pelleted by centrifugation at 2000 ×g for 5 minutes at 4°C. The density was adjusted to 0.1 OD (0.1 OD = 1 × 10^8^ CFUs) in sterile PBS.

### Acute P. aeruginosa infection model

To estimate the severity of pneumonia infected with an intranasal instillation with *P.a*, BALB/c mice were randomly divided into 4 groups (n=6). The Naïve group and the *P.a.* group were sensitized, treated, and challenged with PBS. Other mice were sensitized by intraperitoneal (i.p.) injection with 100 μg HDM absorbed to 2 mg Al (OH) 3 on day 0, 7 and 14. From day 28, mice were received subcutaneous (s.c.) injection (every 2 days, 8 administrations) with PBS (HDM/*P.a.* group) or 100 μg HDM (SIT/*P.a.* group), respectively. The challenge was initiated seven days after the final immunization. Mice were challenged with intranasal (i.n.) instillation of 50 μg HDM daily for 7 days. Twenty-four hours after the last challenge, mice in the *P.a.* group, the HDM/*P.a.* group, and the SIT/*P.a.* group were anesthetized using diethyl ether and were then infected with an intranasal instillation 1×10^7^ CFUs *P.a*.[6]. The Naïve group mice instilled with equivalent doses of PBS. The rectal temperature of mice was measured 24 hours after infection, and then mice were euthanized.

### Measurement of rectal temperature

Rectal temperature was measured using a 1.5-cm thermistor probe. After inserted the probe into the rectum of mice, temperatures were recorded when the probe reading stabilized. Each mouse was triple measured to avoid deviations.

### Histological evaluation

The mice were sacrificed. The cells in the BAL fluid were fixed and stained differential cell counts were performed in duplicate on coded slides for 200 cells from each sample as described previously [39]. The lung tissues were fixed in 10% neutral-buffered formalin and embedded in paraffin. Lung sections were stained with standard hematoxylin-eosin staining (H&E) methods to evaluate the tissue inflammation. The degree of cellular infiltration was scored using previously described methods [40]. A score ranging from 0 to 3 was applied to each observed bronchus, with approximately 10 areas total scored.

### Cytokine assays and HDM specific antibodies levels in sera

The levels of L-4, IL-10, TGF-β1 and IFN-γ in BALF were measured by sandwich enzyme-linked immunosorbent assay (ELISA) with commercial reagent kits (eBbioscience, USA) in accordance with the manufacturer’s instructions. HDM-specific IgE, IgG1 and IgG2a antibodies were measured by indirect ELISA [41]. Briefly, the plates were coated with 100 ng HDM per wells in carbonate buffered solution at 4°C for overnight, blocked at room temperature for 1 hour, and added 100μl serum to each well for 2h. Peroxidase-labeled goat anti-human IgE, IgG1 and IgG2a were added to each well for 1h, and then added 100 μl/well tetramethylbenzidine (TMB) to develop. After stopped by 2M H_2_SO_4_ (50 μl/well), the results were measured by ELx808 absorbance microplate reader (BioTek, Shanghai, China) at 450 nm.

### Evaluation of bacteria in BAL fluid

After BAL fluid were centrifuged (500 ×g for 5 min at 4°C), the precipitate was resuspended and dropped on slides followed by staining with a Gram’s method. The slides were evaluated for bacteria infiltration under a light microscope.

### Quantitation of bacteria in lung

The lungs were removed, weighed, and homogenized in sterile-PBS in aseptic conditions, and aliquots were plated on *P. aeruginosa*-selective plates. Bacterial colonies were counted after incubation at 37°C for 24 hours.

### Cell lines

The SV40-transformed human bronchial epithelial cell line 16HBE14o–was obtained from Xiangya Central Experiment Laboratory (Xiangya School of Medicine, CSU, Changsha, Hunan, China). Cells were cultured in Dulbecco’s modified eagle medium (DMEM; Gibco, Thermo Fisher Scientific) supplemented with 10% heat-inactivated fetal bovine serum (FBS; Gibco, Thermo Fisher Scientific), 2 mM L-glutamine (Gibco, Thermo Fisher Scientific), 100 U/mL penicillin (Gibco, Thermo Fisher Scientific), and 100 μg/mL streptomycin (Gibco, Thermo Fisher Scientific). 16HBE14o– cells were grown to 85–90% confluence and were serum deprived for 18 to 24 hours before treatments.

### Epithelial cell culture

To observe the expression of CRAMP in epithelial cells, 16HBE14o– cells were plated to culture dishes, and were allowed to adhere at 37°C under 5% CO2 in electronic controlled incubator (Thermo Forma, USA). After an initial study with TGF-β_1_(1ng/ml), IFN-γ(200IU), and IL-10(50ng/ml), we found that treatment of cytokines cannot increase the expression of CRAMP in 16HBE14o– cells. In subsequent experiments, the cells were treated with 25VD_3_(10nM), TGF-β_1_(1ng/ml)+25VD3(10nM), IFN-γ(200IU)+25VD3(10nM), IL-10(50ng/ml)+ 25VD3 (10 nM), and 1,25VD_3_(10nM) in serum-free culture medium for 24 h. To evaluate the expression of CYP27b1 in 16HBE14o– cells. We stimulated the cells with TGF-β_1_ in different doses (0.1ng/ml, 1ng/ml, and 10ng/ml) for 24 hours.

### Inhibition of CYP27b1

16HBE14o– cells were prepared as described above and pre-treated with itraconazole (10^−7^ M), a specific inhibitor of CYP27b1, for 2 hours, and then stimulated with TGF-β_1_ (1 ng/ml)+25 VD3 (10 nM) for 24 h. CRAMP levels in cells were determined.

### Cell infection experiments

16HBE14o–cells were changed to antibiotic-free medium. Cells were treated with 25VD_3_(10nM), TGF-β1(1ng/ml), TGF-β_1_(1ng/ml)+25VD3(10nM), 25 VD3(10nM)+ITRA(pretreated with ITRA for 2 h), TGF-β1(1ng/ml)+ 25 VD3(10nM)+ITRA(pretreated with ITRA for 2 h) for 48 hours at 37°C. And parts of cells were only treated with ITRA for 2 h for control. Then cells were infected by *P. aeruginosa* in an MOI of 10:1 bacteria-cell ratio. After incubation for 1 hour, the cells were washed with PBS twice and incubated with fresh medium containing polymyxin 100 μg ml^−1^ to kill extracellular bacteria. After 1 hour, samples were plated in LB solid culture medium to confirm that the extracellular bacteria had been killed. The cells were then homogenized with PBS and spread on LB plates. Bacterial colonies were counted after incubation at 37°C for 24 hours.

### Real-time RT-PCR

Total RNA was extracted from lung tissue or cell lysate with TRIzol Reagent (Invitrogen, Thermo Scientific, USA) as recommended by the manufacturer. A total of 1.5 μg of total RNA preparation was reverse transcribed using a cDNA synthesis kit (RevertAid First Strand cDNA Synthesis Kit, Thermo Scientific, USA). cDNA was diluted 1/5, and 5 μl was used as template in a 50 μl SYBR-Green PCR reaction system, according to the manufacturer’s instruction (iQ™ SYBR® Green, Bio-Rad, USA). β-actin premier(sense, 5’-CATCCGTAAAGACCTCTATGCCAAC-3’; antisense, 5’-ATGGAGCCACCGATCCACA-3’), GAPDH primer(sense, 5’-GGAGTCAACG GATTTGGT CGTA-3’; antisense, 5’-GCAACAATATCCACTTTACCAGAGTTAA-3’), CRAMP primer(Mus musculus) (sense, 5’-GTCTTGGGAACCATGCAGTT-3’; antisense, 5’- TGGTTGAAGTCATCCACAGC-3’), CRAMP primer(Homo sapiens) (sense, 5’-GTCACCAGAGGAT TGTGACTTCAA-3’; antisense, 5’-TTGAGGGTCA CTGTCCCCATA-3’), and CYP27b1 primer(sense, 5’-GTTTGTGTCCACGCTG-3’; antisense, 5’-CCCGC CAATAGCAACT-3’) were synthesized by Sangon Biotech(Shanghai, China). Specificity of RT-PCR was controlled by omission of the template or the reverse transcription. Quantitative PCR results were obtained using the 2-ΔΔCt method and were normalized to β-actin or GAPDH.

### Immunofluorescent staining

Frozen lung tissues at −80°C were sectioned, and were fixed in acetone and blocked at room temperature. Sections were incubated with CRAMP antibody. A mouse isotype serum instead of the primary Abs was used as a negative control. FITC-conjugated goat anti-rabbit Ab(Cat. No.4030-02, SouthernBiotech, USA) as secondary Abs were used to probe the primary Abs. The cell nucleus was stained with PI (Sigma-Aldrich, USA). Tissue sections were viewed with an Olympus BX51 fluorescence microscope (Japan).

### Western blotting

The cells were homogenized with lysis buffer (1000 μl RIPA with 10 μl phenylmethanesulfonyl fluoride (PMSF), Beyotime, China) in ice bath. Protein assay was performed using a bicinchoninic acid (BCA) concentration measurement kit (Solarbio, China) to ensure each sample contained equal amounts of protein. Proteins (20μg/lane) were loaded and then run on 10% sodium dodecyl sulfate-polyacrylamide gel electrophoresis (SDS-PAGE) at 100 V for 90 min. Dissolved proteins were transferred onto a nitrocellulose membrane by electroblotting with Bio-Rad blotting transfer unit(Bio-Rad, USA) at 300mA for 45 min in ice bath. Non-specific binding sites were blocked with 5% skim milk at room temperature for 2 hours. The membranes was incubated overnight at 4°C with rabbit polyclonal antibody against CRAMP (1:1000 diluted with 1% skim milk, PAB13021, Abnova, Taipei), or rabbit polyclonal antibody against CYP27b1 (1:200 diluted with 1% skim milk, sc-67261, Santa Cruz Biotechnology, USA), or rabbit monoclonal antibody against GAPDH (1:1000 diluted with 1% skim milk, Cat. No. 2118, Cell Signaling, USA). After washed three times in TBST for 10 min each, the membranes were then incubated with HRP-conjugated goat anti-rabbit antibody (1:10000 dilution, EarthOx, Millbrae, California, USA) at 37°C for 1 hour and washed five times for 10 minutes each with TBST, and then three times for with TBS for 5 minutes each. Chemiluminescent substrate (Bio-Rad’s Clarity ECL Western Blotting Substrate) was added to expose strips. Strips were semiquantitative analyzed with the Image J 1.48V software.

### Immunohistochemistry

Paraffin sections of lung tissue were deparaffinized and rehydrated. Antigen retrieval was performed with high pressure method in citrate (pH = 6.0) for 3 minutes. Normal goat serum was added to block non-specific binding sites at 37°C for 40 minutes. The sections were incubated with rabbit polyclonal antibody against CYP27b1 (1:100 dilution, sc-67261, Santa Cruz Biotechnology, USA) overnight at 4°C. The secondary antibody working solution (Boster, Wuhan, China) was incubated at 37°C for 40 min. After rinsed with PBS, SABC was then incubated for 30 minutes at 37°C. Finally, the slides were visualized using DAB immunostaining under a light microscope.

### Statistical analysis

Data are presented as mean ±SDEV. Statistical analysis was performed using Student’s t test for comparing two groups and one-way ANOVA for multiple group comparisons. Significant differences in the mean values were defined as p < 0.05. All statistical analyses were done with SPSS 19.0 software.
